# Comparison of the immunogenicity of five COVID‐19 vaccines in Sri Lanka

**DOI:** 10.1111/imm.13535

**Published:** 2022-07-09

**Authors:** Chandima Jeewandara, Inoka Sepali Aberathna, Saubhagya Danasekara, Laksiri Gomes, Suranga Fernando, Dinuka Guruge, Thushali Ranasinghe, Banuri Gunasekera, Achala Kamaladasa, Heshan Kuruppu, Gayasha Somathilake, Jeewantha Jayamali, Deshni Jayathilaka, Helanka Dinesh Kumara Wijayatilake, Pradeep Darshana Pushpakumara, Michael Harvie, Thashmi Nimasha, Shiromi Devika Grace de Silva, Ruwan Wijayamuni, Lisa Schimanski, Pramila Rijal, Jack Tan, Alain Townsend, Graham S. Ogg, Gathsaurie Neelika Malavige

**Affiliations:** ^1^ Allergy Immunology and Cell Biology Unit, Department of Immunology and Molecular Medicine University of Sri Jayewardenepura Nugegoda Sri Lanka; ^2^ Ministry of Health Colombo Sri Lanka; ^3^ Colombo Municipal Council Colombo Sri Lanka; ^4^ MRC Human Immunology Unit, MRC Weatherall Institute of Molecular Medicine University of Oxford Oxford UK; ^5^ Centre for Translational Immunology, Chinese Academy of Medical Sciences Oxford Institute University of Oxford Oxford UK

**Keywords:** antibodies, COVID‐19, memory, vaccination

## Abstract

To determine the antibody responses elicited by different vaccines against SARS‐CoV‐2, we compared antibody responses in individuals 3 months post‐vaccination in those who had received different vaccines in Sri Lanka. Abs to the receptor binding domain (RBD) of the ancestral (wild type) virus (WT) as well as to variants of concern (VoCs), and ACE2 blocking Abs, were assessed in individuals vaccinated with Moderna (*n* = 225), Sputnik V (*n* = 128) or Sputnik light (*n* = 184) and the results were compared with previously reported data on Sinopharm and AZD1222 vaccinees. A total of 99.5% of Moderna, >94% of AZD1222 or Sputnik V and >70% of Sputnik light, >60% of Sinopharm vaccine recipients, had a positive response to ACE2 blocking antibodies. The ACE2 blocking antibody levels were highest to lowest was Moderna > Sputnik V/AZD1222 (had equal levels) > Sputnik light > Sinopharm. All Moderna recipients had antibodies to the RBD of WT, alpha and beta, while positivity rates for delta variant was 80%. The positivity rates for Sputnik V vaccinees for the WT and VoCs were higher than for AZD1222 vaccinees while those who received Sinopharm had the lowest positivity rates (<16.7%). The total antibodies to the RBD were highest for the Sputnik V and AZD1222 vaccinees. The Moderna vaccine elicited the highest ACE2 blocking antibody levels followed by Sputnik V/AZD1222, while those who received Sinopharm had the lowest levels. These findings highlight the need for further studies to understand the effects on clinical outcomes.

## Graphical abstract

We found that the seropositivity rates, ACE2 blocking antibody levels and antibodies to the receptor binding domain of variants of concern showed a significant variation between vaccines. The levels of ACE2 blocking antibodies were the highest for Moderna, followed by Sputnik V and AZD1222, followed by the lowest for Sinopharm. These differences in the antibody response to different vaccines may have significant implications in breakthrough infection rates, hospitalization and severe disease in different vaccine recipients.

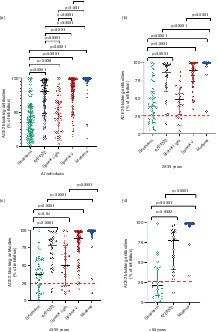

## INTRODUCTION

With the emergence and rapid spread of the SARS‐CoV‐2 omicron variant, many high income and upper middle‐income countries have ramped up their vaccination programmes by rolling out booster doses to all individuals over 18 years of age [1], while many individuals in lower income countries are yet to receive their first dose [2]. There are currently seven COVID‐19 vaccines which the WHO has given emergency use authorization [3], while some other vaccines such as Gam‐COVID‐Vac (Sputnik V and Sputnik light) have been widely used without WHO emergency use license [4]. The United States, Europe and other high‐income countries have vaccinated their populations largely either using an mRNA vaccines (mRNA‐1273 or BNT162b2) or AZD1222, while many lower middle‐income countries and low‐income countries have been using inactivated vaccines such as Sinovac, Sinopharm (BBIBP‐CorV) or the adenovirus vector vaccine Gam‐COVID‐Vac [4].

The effectiveness of the different COVID‐19 vaccines varies widely, and the levels of neutralizing antibodies (Nabs) elicited by different vaccines have shown to correlate with efficacy rates [5]. A direct comparison between four different vaccines showed that 2 to 3 months post immunization showed that the Pfizer‐BioNTech (BNT162b2), elicited the highest levels of ACE2 blocking antibodies, followed by AZD1222, Sputnik V and Sinopharm [6]. Furthermore, the waning of Nabs and T cell responses with time has been shown to vary widely for different vaccines [7, 8, 9]. Differences in the induction of Nabs and their persistence is likely to have a significant impact on the transmission dynamics of the SARS‐CoV‐2 variants of concern (VoC), especially with the emergence of the omicron variant. A 41‐fold decline in Nabs elicited by the Pfizer‐BioNTech vaccine and 30‐ to 60‐fold reduction of Nabs in convalescent plasma was observed for the omicron variant [10, 11]. This immune escape by omicron was found to be less in those who were previously infected and vaccinated [11]. In order to prepare for the rapid spread of omicron globally, many high‐income countries have reduced the gap between the 2nd dose and the booster to 3 months and have decided to give a booster dose to all adults over 18 years of age [12, 13]. Although it appears that the omicron variant significantly evades immunity, induction of higher Nabs through giving a booster dose, is likely to reduce this immune escape [11]. However, the Nabs levels following booster doses would depend on the Nabs levels post second dose. Furthermore, although high‐income countries are rapidly deploying booster doses and therefore could possibly reduce the impact due to the rapid transmission of omicron, the transmission dynamics and clinical disease severity could be different in many lower middle‐income countries with lower infection rates, and lower vaccination rates.

Sri Lanka has currently fully vaccinated 64% of its population, while 74% have received at least a single dose of the vaccine [4]. Sinopharm (BBIBP‐CorV) was the main vaccine used with 11.9 million individuals [14], which is 56.9% of the total population. Some individuals were also vaccinated with Sputnik V and due to the late arrival of the second dose of Sputnik V, many individuals only received the first dose of the Gam‐COVID‐Vac (rAd26‐S/Sputnik light), which is marketed as a single dose vaccine. We had previously published, and data on the kinetics of antibody and T cell responses to the AZD1222 [9] and the Sinopharm vaccine in the Sri Lankan population, which showed significant differences. In order to get a better idea regarding the differences in immunogenicity of different vaccines, we wished to build on that data, by carrying out a direct comparison for the immunogenicity of different vaccines, at the same time point post‐vaccination. Therefore, we compared the total antibody levels (IgG, IgA and IgM) and NAbs to the receptor binding domain (RBD) of the SARS‐CoV‐2 ancestral strain and VOCs, 3 months post vaccination, in individuals who received the Moderna, AZD1222, Sinopharm, Sputnik V, or Sputnik light vaccines. We further compared the antibody levels in vaccinees who were uninfected and who were naturally infected to determine the impact of natural infection on the antibody levels with natural infection. We have measured the Nabs using two surrogate assays neutralization assays, which are the surrogate virus neutralization test (sVNT) and a haemagglutination test (HAT) which were shown to be highly specific in measuring Nabs to SARS‐CoV‐2 virus in the Sri Lankan population [15, 16].

## METHODS

### Study participants

Sri Lankan individuals who had received either the AZD1222 (AstraZeneca/Covishield) vaccine, Moderna (mRNA‐1273), Sinopharm/BBIBP‐CorV, Sputnik light or Sputnik V, were recruited 3 months from receiving the second dose of the vaccine, following informed written consent. These individuals were initially recruited when they received the first dose of the respective vaccines, at the vaccination centres in Sri Lanka and a followed‐up blood sample was taken at 3 months since obtaining the second dose of the vaccine. In those who received Sputnik light, blood samples were taken 3 months post‐vaccination. The data for individuals who received AZD1222 (12 weeks gap between the two doses) [9] and the Sinopharm/BBIBP‐CorV [8] have been previously described. These data on antibody responses to these two vaccines we described in these two studies were then used to compare the immunogenicity of these two vaccines with Moderna and Sputnik light and Sputnik V.

In those who received the Moderna, AZD1222 or Sputnik (one or two doses), those who had antibodies to the N protein of the SARS‐CoV‐2 virus were considered as being infected and were excluded from the analysis. Details of the N protein assay is described below. The demographic features of the individuals who were included in the comparison of the immunogenicity of different vaccines are shown in Table 1. We could not recruit individuals >60 years to study the immunogenicity of the Sputnik vaccines, as this vaccine was not given to those >60 years of age in Sri Lanka based on a policy decision by the Ministry of Healthy, Sri Lanka. Therefore, the immunogenicity for these vaccines was only assessed in individuals <60 years of age.

**TABLE 1:** Percentage of individuals seroconverted based on total RBD antibodies and ACE2 blocking antibodies for SARS CoV‐2 at 3 months following full vaccination

Vaccine	Antibody type	Percentage positivity (number of positive individuals/total number) median age (range)			
		All individuals mean age (range)	Age 20 to 39	Age 40 to 59	Age >60
AZD1222	RBD‐Abs	100% (299/299) 45 years (21–81)	100% (129/129)	100% (152/152)	100% (18/18)
	ACE2 blocking‐Abs	96.2% (65/69) 49 years (24–81)	96.1% (25/26)	92.3%% (24/26)	94.1% (16/17)
Sinopharm	RBD‐Abs	95.07% (193/203) 47 years (25–72)	96.72% (59/61)	95.0% (114/120)	90.91% (20/22)
	ACE2 blocking‐Abs	60.9% (67/110) 47 years (25–72)	65.8% (27/41)	66.6% (32/48)	38.1% (8/21)
Moderna	RBD‐Abs	94.2% (214/227) 50 years (22–82)	96% (48/50)	93.7% (121/129)	93.7% (45/48)
	ACE2 blocking‐Abs	99.5% (223/224) 50 years (22–82)	100% (48/48)	99.2% (131/132)	100% (44/44)
Sputnik Light (1 dose)	RBD‐Abs	94.5% (174/184) 46 years (20–59)	95.5% (43/45)	94.2% (131/139)	0
	ACE2 blocking Abs	74% (37/50) 40 years (20–59)	76% (19/25)	72% (18/25)	0
Sputnik V (2 doses)	RBD‐Abs	100% (127/127) 44 years (25–66)	100% (50/50)	100% (77//77)	0
	ACE2 blocking‐Abs	97.7% (124/127)	100% (50/50)	96.1% (74/77)	0

Testing for antibodies in individuals who were found to be naturally infected prior to vaccination were analysed separately. Antibodies to the S protein (RBD specific antibodies) of the virus was carried out in all individuals in blood samples obtained at the time of recruitment (when they received the first vaccine dose of the respective vaccine). Individuals who were found to have antibodies to the RBD of the S protein by the Wantai SARS‐CoV‐2 antibody ELISA, were considered to have been naturally infected. The N protein specific antibody tests were carried out in the blood samples obtained at 3 months post first dose in those who received the AZD1222, Moderna or Sputnik V or Sputnik light vaccine and if positive they were considered to have been naturally infected within the 3 months. Those who reported a symptomatic COVID‐19 infection and tested positive were also considered to be naturally infected.

Ethics approval was obtained from the Ethics Review Committee of University of Sri Jayewardenepura.

### Detection of total antibodies to the RBD of SARS‐CoV‐2

SARS‐COV‐2 specific total antibody (IgM, IgG and IgA) responses to the RBD were assessed using the Wantai SARS‐CoV‐2 antibody ELISA (Beijing Wantai Biological Pharmacy Enterprise) as previously described according to the manufacturer's instructions [17]. The antibody index (an indirect measure of the total antibody levels to the RBD) was calculated by dividing the absorbance of each sample by the cutoff value, according to the manufacturer's instructions. This assay was shown to have a sensitivity of 98% and was found to be 100% specific when tested using serum samples obtained in 2018, in Sri Lankan individuals [18].

### Measuring the presence of neutralizing antibodies to the SARS‐CoV‐2 using a surrogate assay

A sVNT [19], which measures the percentage of inhibition of binding of the RBD of the spike protein to recombinant ACE2 (Genscript Biotech) was used to measure the ACE2 blocking antibodies. Inhibition percentage ≥25% in a sample was considered as positive for ACE2 blocking antibodies. This assay was found to be 100% specific for measuring ACE2 blocking antibodies in the Sri Lankan population [15]. The sVNT was only done in a sub cohort of individuals for each of the vaccines. The number of individuals investigated according to age groups are shown in Table 1.

### Assays to determine antibodies to the N protein

Qualitative detection of antibodies to SARS‐CoV‐2 nucleocapsid (N) antigen was carried out using the Elecsys® Anti‐SARS‐CoV‐2 electrochemiluminescence immunoassay (Cat: 09203095190, Roche Diagnostics) using the Cobas e 411 analyser (Roche Diagnostics). A cutoff index (COI) ≥1.0 was interpreted as reactive and COI <1.00 was considered non‐reactive as per the kit guidelines.

### HAT to detect antibodies to the RBD of VOCs

The in‐house HAT developed by Townsend at al [20], was carried out as previously described using the B.1.1.7 (N501Y), B.1.351 (N501Y, E484K, K417N) and B.1.617.2 versions of the IH4‐RBD reagents [20], which included the relevant amino acid changes introduced by site directed mutagenesis. The assays were carried out and interpreted as previously described and a titre of 1:20 was considered as a positive response [16, 17]. The HAT titration was performed using seven doubling dilutions of serum from 1:20 to 1:1280, to determine presence of antibodies to the RBD in the different VOCs. The RBD‐specific antibody titre for the serum sample was defined by the last well in which complete absence of ‘teardrop’ formation was observed. A titre of 1:20 was considered as a positive response, as previously determined [16].

### Statistical analysis

The analysis of data was conducted using the R software (version 4.0.3), R‐Studio (version 1.4.1106) and GraphPad PRISM version 8.3. As the data were not normally distributed, differences in the antibody levels measured by different assays for different vaccines were compared using the Mann–Whitney *U* test (two tailed).

## RESULTS

### Seroconversion rates for the different vaccines

**FIGURE 1:**
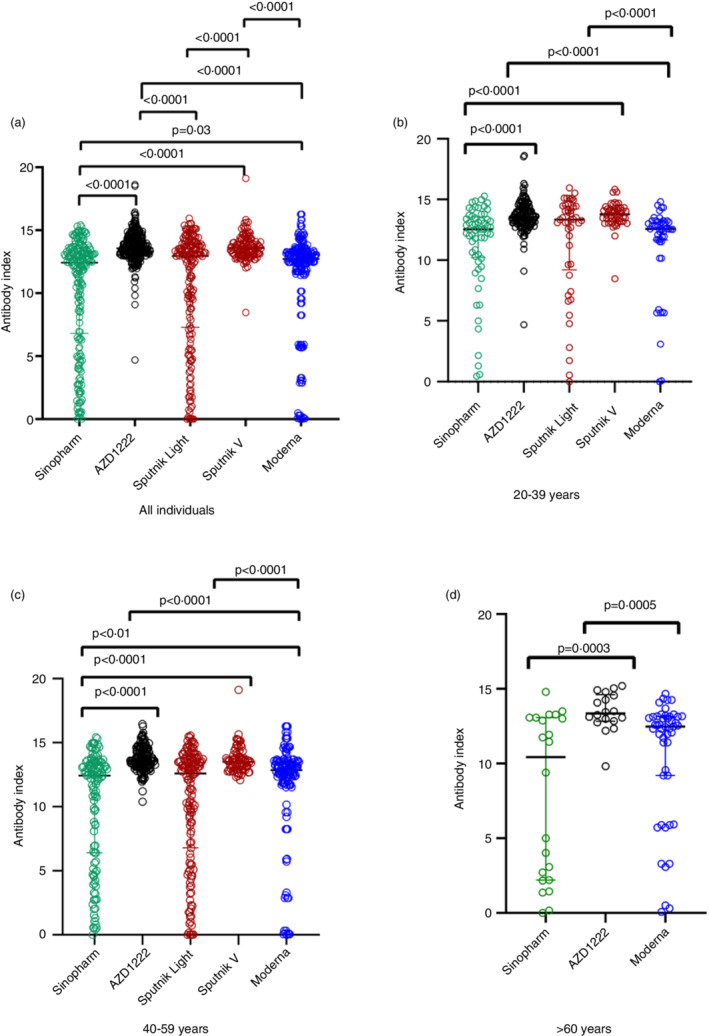
SARS‐CoV‐2 specific total antibodies to the receptor binding domain (RBD) in those who received different vaccines at 3 months following full vaccination. Total antibodies to the RBD were measured by ELISA in all individuals who received two doses of Sinopharm (*n* = 203), AZD1222 (299), Sputnik 1 dose (Sputnik light, *n* = 184), Sputnik 2 doses (Sputnik V, *n* = 127) and Moderna 2 dose (*n* = 227) (a). Total antibodies to the RBD were also measured in 20‐ to 39‐year‐olds who received two doses of Sinopharm (*n* = 61), AZD1222 (129), Sputnik 1 dose (*n* = 45), Sputnik 2 doses (*n* = 50) and Moderna 2 doses (*n* = 50) (b). Total antibodies to the RBD were also measured in 40 to 59‐year‐olds who received two doses of Sinopharm (*n* = 120), AZD1222 (152), Sputnik 1 dose (*n* = 139), Sputnik 2 doses (*n* = 77) and Moderna 2 doses (*n* = 129) (c). In those >60 years of age, the analysis was carried out in those who received 2 doses of Sinopharm (*n* = 22), 2 doses of AZD1222 (*n* = 18) and 2 doses of Moderna (*n* = 48) (d). The differences in antibody titres (antibody index) between different vaccines were analysed using the Mann–Whitney test. All tests were two‐tailed. The lines indicate the median and the inter quartile range. Only *P* values which indicate a significant difference are shown in the figure.

The number of individuals who gave a positive response for the total antibody responses (IgG, IgA and IgM) to the RBD of the virus, and ACE2 receptor blocking antibodies for the entire cohort and for different age groups, by vaccine type is given in Table 1 and Figure 1a. As we have previously shown, all those who received the two doses of the AZD1222 vaccine had seroconverted [9], whereas the overall seroconversion rate for Sinopharm was 95.07% [8]. All individuals (100%) who had received both doses of the Sputnik vaccines, 94.2% of individuals who received Moderna and 94.5% of those who received Sputnik light had seroconverted based on the presence of RBD binding total antibodies measured by the Wantai assay. In contrast, the positivity rates for ACE2 blocking antibodies (surrogate measure for the presence of Nabs) was highest for Moderna (99.5%), followed by Sputnik V (97.7%).

### Total antibody responses to the SARS‐CoV2 RBD

In individuals in the 20 to 39 age group and 40 to 59 age group, those who received 2 doses of AZD1222 and two doses of Sputnik V had significantly higher (*P* < 0.0001) total antibody responses to the RBD than those who received two doses of the Sinopharm vaccine, 2 doses of Moderna (*P* < 0.0001) and Sputnik light (*P* < 0.0001) (Figure 1b,c). In addition, individuals who received Moderna had significantly higher responses (*P* = 0.01) than those who received both doses of Sinopharm (Figure 1c).

In those who were >60 years of age, the analysis was only carried out for Sinopharm, AZD1222 and Moderna as individuals >60 years of age did not receive the Sputnik vaccines. Those who had received 2 doses of AZD1222 had significantly higher total antibody responses to the RBD than those who had received both doses of Sinopharm (*P* = 0.0003) and both doses of Moderna (*P* = 0.0005) (Figure 1d). There was no difference in the total Ab levels to the RBD between those who had received Sinopharm compared to Moderna (*P* = 0.09) in this age group.

### ACE2 blocking antibodies assessed by the surrogate neutralizing antibody test (sVNT) for different vaccines

**FIGURE 2:**
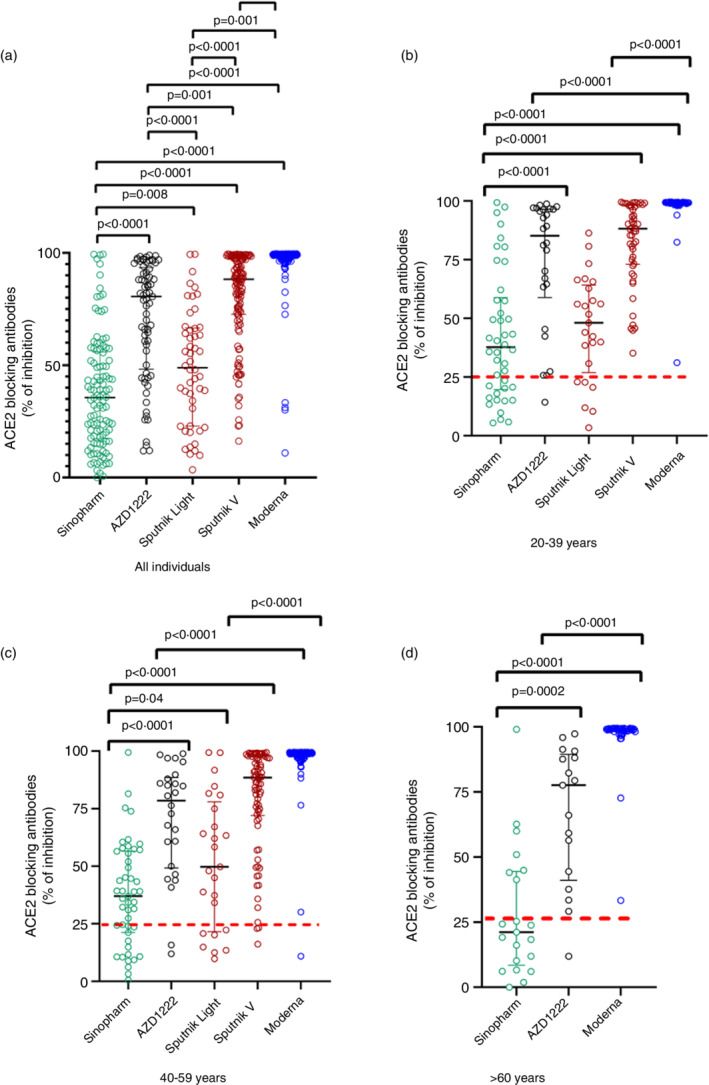
SARS‐CoV‐2 ACE2 blocking antibodies in those who received different vaccines at 3 months following full vaccination. ACE2 blocking antibodies were measured by the sVNT assay in 20‐ to 39‐year‐olds who received two doses of Sinopharm (*n* = 110), AZD1222 (224), Sputnik 1 dose (Sputnik light, *n* = 50), Sputnik 2 doses (Sputnik V, *n* = 50) and Moderna 2 doses (*n* = 48) (a). ACE2 blocking antibodies were also measured in 20‐ to 39‐year olds who received two doses of Sinopharm (*n* = 41), AZD1222 (26), Sputnik 1 dose (*n* = 25), Sputnik 2 doses (*n* = 50) and Moderna 2 doses (*n* = 48) (b). ACE2 blocking antibodies were also measured in 40‐ to 59‐year‐olds who received two doses of Sinopharm (*n* = 48), AZD1222 (26), Sputnik 1 dose (*n* = 25), Sputnik 2 doses (*n* = 77) and Moderna 2 doses (*n* = 132) (c). In those >60 years of age, the analysis was carried out in those who received 2 doses of Sinopharm (*n* = 21), 2 doses of AZD1222 (*n* = 17) and 2 doses of Moderna (*n* = 44) (d). The differences in ACE2 blocking antibodies (% of inhibition) between different vaccines were analysed using the Mann–Whitney test. All tests were two‐tailed. The lines indicate the median and the inter quartile range. The positive cut‐off value if shown as a red dotted line. Only *P* values which indicate a significant difference are shown in the figure.

ACE2 blocking antibodies were assessed in a sub cohort of previously uninfected individuals for each vaccine and the overall ACE2 blocking antibody levels for different vaccines is shown in Figure 2a. In the 20 to 39 age group and 40 to 59 age group, those who received two doses of AZD1222, Sputnik V and Moderna had significantly higher (*P* < 0.0001) ACE2 blocking antibodies than those who received two doses of Sinopharm or one dose of Sputnik (Figure 2b,c). In contrast to what was observed with the SARS‐CoV‐2 total antibodies to the RBD, those who received two doses of Moderna had significantly higher (*P* < 0.0001) ACE2 blocking antibodies than those who had two doses of AZD1222 or Sputnik V (Figure 2b,c). In the 20 to 39 age group, the median ACE2 blocking antibody levels for Moderna was 99.2% (IQR 98.8 to 99.4% of inhibition), while levels following Sputnik V were 88.2 (IQR 73.1 to 98.1% of inhibition), for AZD1222 were 85.2 (IQR 58.9 to 96.5% of inhibition) and for Sinopharm 37.7 (IQR 19.6 to 58.9% of inhibition) as previously reported [8, 21].

In the 40 to 59 age group, the median ACE2 blocking antibody levels for Moderna was 98.9% of inhibition (IQR 98.9 to 99.3% of inhibition), while levels following two doses of Sputnik V was 88.4 (IQR 72.0 to 98.6% of inhibition) the levels for AZD1222 were 78.5% inhibition (IQR 49.1 to 88.4% of inhibition) and for Sinopharm 37.0% (IQR 21.2 to 56.5% of inhibition) as we previously showed [8, 21].

The analysis in individuals above 60 years of age was limited to Sinopharm, AZD1222 and Moderna. The ACE2 blocking antibodies were significantly higher in those who received 2 doses of AZD1222 (*P* = 0.0002) and Moderna (*P* < 0.0001) compared to those who received Sinopharm (Figure 2d). The ACE2 blocking antibody levels were also significantly higher (*P* < 0.0001) in those who received Moderna compared to those who received AZD1222 (Figure 2d). The median ACE2 blocking antibody levels for Moderna was 99.0% (IQR 98.2 to 99.4% of inhibition), while the levels for AZD1222 were 77.6 (IQR 41.0 to 89.4% of inhibition) and for Sinopharm 21.1 (IQR 8.4 to 44.5% of inhibition) as we have previously shown [8, 9].

### SARS‐CoV‐2 RBD specific antibodies measured by the haemagglutination assay (HAT) for the ancestral virus and the VoCs

As the vaccines given Moderna had significantly higher levels of ACE2 blocking antibodies than those who received the two adenovirus vector vaccines, AZD1222 and Sputnik V, we proceeded to investigate the differences in the antibody responses to RBD of the SARS‐CoV‐2 and VOCs by using the HAT assay, as this assay too has shown to correlate with neutralizing antibodies [22]. The positivity rates in those who received two doses of Moderna and two doses of Sputnik V in different age groups is shown in Table 2. Similar to the results seen with the sVNT assay, all those who received two doses of Moderna had a positive response to the SARS‐CoV‐2 ancestral strain (WT), alpha and beta variants, whereas positivity rates were 84% in the 20 to 39 and 40 to 59 age groups for delta. In contrast, the positivity rates for the WT and VOC in those who received two doses of Sputnik V were between 68% and 80%. Although these positivity rates for Sputnik V were higher than those seen following two doses of AZD1222 (50% to 65%) in 20 to 39 and 40‐to‐59‐year age groups [9], the positivity rates were lower than those following Moderna.

**TABLE 2:** Detection of antibodies specific for the RBD of the SARS‐CoV‐2 ancestral strain (WT) and variants of concern (VoCs) in individuals vaccinated with Moderna and Sputnik V measured by the haemagglutination test (HAT) at 3 months following vaccination

Vaccine type	Age groups	Percentage positivity (number of positive individuals/total number)			
		WT	B.1.1.7 (alpha)	B.1.351 (beta)	B.1.617.2 (delta)
Moderna	20 to 39	100% (26/26)	100% (26/26)	100% (26/26)	84.6% (22/26)
	40 to 59	100% (25/25)	100% (25/25)	100% (25/25)	84% (21/25)
	>60	100% (13/13)	100% (13/13)	100% (13/13)	100% (13/13)
Sputnik V	20 to 39	80% (20/25)	68% (17/25)	68% (17/25)	76% (19/25)
	40 to 59	80.4% (37/46)	71.7% (33/46)	67.3% (31/46)	73.9% (34/46)

**FIGURE 3:**
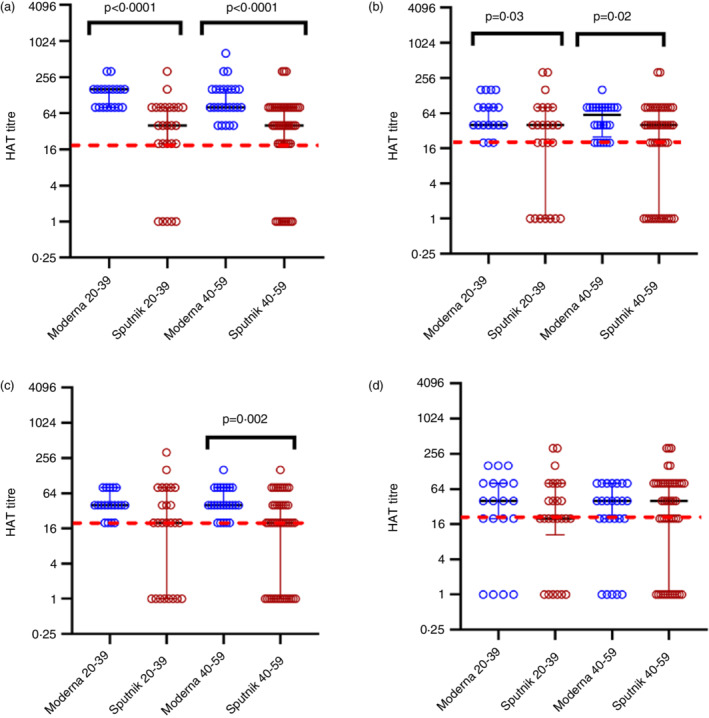
SARS‐CoV‐2 specific antibodies to the receptor binding domain (RBD) of the ancestral (WT) virus and variants of concerns in those who received Sputnik V and Moderna 3 months following the second dose. Antibodies to the RBD were measured by the haemagglutination test (HAT) in those who received two doses of Moderna in 20‐ to 39‐years‐old (*n* = 26) or two doses of Sputnik V (*n* = 25) and those who were aged 40 to 59 years who received two doses of Moderna (*n* = 25) or Sputnik V (*n* = 46). Antibodies were measured by HAT to the WT (a), B.1.1.7 (b), B.1.351.1 (c) and B.1.617.2 (d). The differences between HAT titres for between the two vaccines were analysed using the Mann–Whitney test. All tests were two‐tailed. The lines indicate the median and the inter quartile range. The positive cut‐off value if shown as a red dotted line. Only *P* values which indicate a significant difference are shown in the figure.

The HAT titres for the WT were significantly higher following Moderna compared to both doses of Sputnik V in 20 to 39 and 40 to 59 age groups (*P* < 0.0001) (Figure 3a). HAT titres were also significantly higher for B.1.1.7 in the 20 to 39 (*P* = 0.03) and the 40 to 59 (*P* = 0.02) age groups for Moderna compared to Sputnik V although the difference was less than for the WT (Figure 3b). For B.1.351, a significant difference between HAT titres was only seen in the 40 to 59 age group, with those who received Moderna having significantly higher (*P* = 0.002) levels (Figure 3c) whereas no difference was seen in the HAT titre levels for B.1.617.2 between either vaccine for any of the two age groups (Figure 3d).

### SARS‐CoV‐2 total antibody responses and ACE2 blocking antibodies in those who were naturally infected prior to vaccination

In order to determine the immune responses in those who had natural infection and the different vaccines, we compared to immune responses of those who were fully vaccinated with one of the COVID‐19 vaccines used in Sri Lanka (those who received the first dose of Sputnik were also investigated separately). The SARS‐CoV‐2 total antibodies and the ACE2 blocking antibody positivity and levels are shown in Table 3. As infected individuals in each group, who received the different vaccines was small, the analysis of the Ab levels was not performed per age group.

**TABLE 3:** The positivity rates and median antibody titres for the total RBD and ACE2 blocking antibodies for SARS‐CoV‐2 in uninfected and infected vaccine recipients at 3 months following full vaccination

Vaccine	Antibody type	Number of positive individuals/total number = % positive median (IQR)		*P* value
		Uninfected individuals	Infected individuals	
AZD1222	RBD‐Abs (Antibody Index)	297/297 = 100% 13.6 (13.1–14.3)	28/28 = 100% 13.7 (13.3–14.6)	0.117
	ACE2 blocking‐Abs (% of inhibition)	66/69 = 95.7% 80.6 (56.4–92.9)	26/28 = 92.9% 75.1 (56.3–96.0)	0.87
Moderna	RBD‐Abs (Antibody Index)	212/224 = 94.6% 12.7 (11.7–13.3)	36/40 = 90.0% 12.9 (11.9–13.3)	0.574
	ACE2 blocking‐Abs (% of inhibition)	223/224 = 99.6% 99.05 (98.4–99.3)	40/40 = 100% 99.2 (99.1–99.4)	0.24
Sinopharm	RBD‐Abs (Antibody Index)	193/203 = 95.1% 12.4 (6.9–13.4)	33/33 = 100% 13.5 (13.1–13.8)	<0.0001
	ACE2 blocking‐Abs (% of inhibition)	67/110 = 60.9% 35.6 (17.7–54.8)	31/33 = 93.9% 67.0 (44.8–83.2)	<0.0001
Sputnik Light (1 dose)	RBD‐Abs (Antibody Index)	174/184 = 94.6% 13.0 (7.4–13.8)	33/33 = 100% 13.6 (12.8–14.3)	0.002
	ACE2 blocking‐Abs (% of inhibition)	37/50 = 74.0% 48.9 (24.9–66.2)	11/12 = 91.7% 99.3 (89.7–99.4)	0.001
Sputnik V (2 doses)	RBD‐Abs (Antibody Index)	128/128 = 100% 13.6 (13.1–14.1)	31/31 = 100% 13.6 (13.0–14.6)	0.984
	ACE2 blocking‐Abs (% of inhibition)	125/128 = 97.7% 88.3 (73.1–97.6)	31/31 = 100% 99.3 (99.1–99.4)	<0.0001

As shown in Table 3, the seropositivity rates for the RBD of the SARS‐CoV‐2 (total antibody levels) and ACE2 blocking antibodies were both significantly higher in infected individuals 3 months post‐immunization, in those who received two doses of Sinopharm or one dose of Sputnik. For those who received two doses of Moderna, AZD1222 or Sputnik V, there was no difference in the total antibody levels to the RBD. However, the levels of ACE2 blocking antibodies were significantly higher in infected individuals following two doses of Sputnik V vaccines, compared to uninfected vaccinees, whereas no difference was seen between uninfected and infected individuals who received AZD1222 or Moderna.

## DISCUSSION

In this study, we have compared the total antibody levels to the RBD of the SARS‐CoV‐2, antibody levels to the RBD of VoCs and ACE2 blocking antibodies in those who received AZD1222, Moderna, Sinopharm, Sputnik V or Sputnik light, 3 months post‐immunization analysed at a single centre in Sri Lanka based on published and new analyses [8, 9]. We found that 99.5% of those in all age groups who received Moderna had ACE2 blocking antibodies, whereas the positivity rates for those who received two doses of AZD1222, or Sputnik V was over 94%. In contrast, the positivity rates following the sputnik light was 74% and as we previously reported for Sinopharm it was 60.9% [8]. Those who received Moderna also had significantly higher levels of ACE2 blocking antibodies than those who were given other vaccines, followed by two doses of AZD1222 or Sputnik V as reported previously [23]. Nab levels have shown to strongly correlate with the level of protection against symptomatic COVID‐19 [5].

Nab levels have also shown to be a correlate of vaccine efficacy and booster doses were shown to increase the vaccine efficacy by increasing Nab titres [24]. The surrogate Nab test (sVNT) that measures ACE2 blocking antibodies has been widely used as a surrogate measure for Nabs [19, 25, 26]. It has been shown that Nabs for current COVID‐19 vaccines vary by as much as 25‐fold [5]. Our data show significant differences months post‐vaccination with different vaccines. Waning of immunity following Moderna and AZ has been associated with breakthrough infections, including an increase in hospitalization rates, especially after 20 weeks [27] although waning of efficacy was lower following Moderna [28]. A reduction in breakthrough infections and hospitalizations has been shown to correlate with Nabs following booster doses of the BNT162b2 vaccine [29, 30]. Although there are no data regarding the effectiveness of Sinopharm, Sputnik light and Sputnik V in preventing breakthrough infections, hospitalizations and severe disease, those who received Sinopharm and Sputnik light had substantially less ACE2 blocking antibodies than those who received the other vaccines. Sinopharm is whole virus vaccine and therefore, induces immune responses beyond spike which may be relevant, and were not tested here. We have not analysed T cell responses which may also impact, but overall the differences detected here are may be relevant particularly with the emergence of the omicron variant, which has a potential to further evade vaccine immunity [31].

Although the Moderna induced the highest levels of ACE2 blocking antibodies in all age groups compared to other vaccines, the total antibody levels measured by the commercial Wantai antibody assay, which detects IgM, IgG and IgA antibodies to the RBD of the virus were significantly lower compared to those who received either two doses of AZD1222 or Sputnik V. In fact, 4.2% to 12.5% of those who received the Moderna vaccine did not have detectable antibodies to the RBD, whereas those who received two doses of the adenovirus vector vaccines had significantly higher levels. Since the findings based on this assay were different to those of the sVNT assay, we compared the antibodies to the RBD of the WT and VoCs by the (HAT), which was shown to correlate with the Nabs and with the sVNT, in those who received two doses of Moderna or Sputnik V [16, 22]. With the HAT assay again, all (100%) those who received Moderna had a positive response to WT, B.1.1.7 and B.1.351.1, and 84 to 100% to B.1.617.2, higher than the positivity rates for Sputnik V and AZD1222 [9]. Since the HAT and the sVNT were shown to strongly correlate with Nabs levels [19, 22], it appears that while those who received Moderna had significantly higher Nabs than those who received other vaccines, those who received the two adenovirus vector vaccines had higher antibody levels to the RBD. While the reasons for these differences are not clear, it could be due to mRNA vaccines having stabilizing substitutions in spike protein to maintain the pre‐fusion conformation, whereas AZD1222 and Sputnik may not contain these specific substitutions [32, 33]. Indeed, it was recently shown that only antibodies that bind the conformational RBD epitopes have a neutralizing capacity while antibodies that bind the linear epitopes were non‐neutralizing [34]. Therefore, it would be important to conduct a prospective study to understand if these differences in antibody levels and positivity rates observed with different assays translate to risk of infection or clinical disease severity.

Although we found that the RBD binding antibodies were highest in those who received AZD1222 and Sputnik V, followed by Moderna, while those who received Sinopharm had the lowest responses, other studies have shown different results [35]. Lijeskic et al. showed that the highest levels of RBD antibodies were seen following the BNT‐162b2 (BioNTech/Pfizer) and Sputnik V, followed by Sinopharm and then one dose of AZD1222 [35]. Although they reported that only 75% of those who received one dose of AZD1222 had RBD binding antibodies, our previous data showed that 93.4% had RBD binding antibodies following a single dose [17].

With the emergence of omicron variant many high‐income countries have now focused their vaccination programmes in rapidly rolling out booster doses [1]. While some countries only gave one dose of a COVID‐19 vaccine to those who had been previously had COVID‐19 [36], there has not been guidance by many authorities in the need of booster doses for those who were fully vaccinated and infected. Our data show that in those who were infected before vaccination, those who recovered from the infection prior to vaccination with either the Moderna or AZD1222, there was no difference in the ACE2 blocking antibodies in infected individuals compared to those who were not previously infected, whereas for Sinopharm and Sputnik the ACE2 blocking antibodies were significantly higher in those who were previously infected. Therefore, infected individuals who received Sinopharm or Sputnik V could benefit from receiving a booster dose of the vaccine.

We found that the seropositivity rates, ACE2 blocking antibody levels and antibodies to the RBD of VOCs showed a significant variation between vaccines. The levels of ACE2 blocking antibodies were the highest for Moderna, followed by Sputnik V and AZD1222, followed by the lowest for Sinopharm. These differences in the antibody responses to different vaccines may have significant implications in breakthrough infection rates, hospitalization and severe disease in different vaccine recipients.

## FUNDING INFORMATION

We are grateful to the Allergy, Immunology and Cell Biology Unit, University of Sri Jayewardenepura; the NIH, USA (grant number 5U01AI151788‐02), UK Medical Research Council and the Foreign and Commonwealth Office for support. T.K.T. was funded by the Townsend‐Jeantet Charitable Trust (charity number 1011770) and the EPA Cephalosporin Early Career Researcher Fund. A.T. was funded by the Chinese Academy of Medical Sciences (CAMS) Innovation Fund for Medical Science (CIFMS), China (grant no. 2018‐I2M‐2‐002).

## CONFLICT OF INTEREST

None of the authors have any conflicts of interest.

## PERMISSION TO REPRODUCE MATERIAL FROM OTHER SOURCES

Not applicable. This manuscript does not contain any material from other sources.

## ETHICS STATEMENT

Ethical approval was received by the Ethics Review Committee of Faculty of Medical Sciences, University of Sri Jayewardenepura. Informed written consent was obtained from patients.

## PATIENT CONSENT STATEMENT

All individuals who participated in the study gave informed written consent.

## CLINICAL TRIAL REGISTRATION

Not applicable as this is not a clinical trial.

## Data Availability

All data are available in the manuscript and the figures.
